# Efficient estimation of SNP heritability using Gaussian predictive process in large scale cohort studies

**DOI:** 10.1371/journal.pgen.1010151

**Published:** 2022-04-20

**Authors:** Souvik Seal, Abhirup Datta, Saonli Basu

**Affiliations:** 1 Department of Biostatistics and Informatics, University of Colorado Anschutz Medical Campus, Aurora, Colorado, United States of America; 2 Department of Biostatistics, Johns Hopkins Bloomberg School of Public Health, Baltimore, Maryland, United States of America; 3 Department of Biostatistics, University of Minnesota, Minneapolis, Minnesota, United States of America; Hasso Plattner Institute for Digital Engineering, GERMANY

## Abstract

With the advent of high throughput genetic data, there have been attempts to estimate heritability from genome-wide SNP data on a cohort of distantly related individuals using linear mixed model (LMM). Fitting such an LMM in a large scale cohort study, however, is tremendously challenging due to its high dimensional linear algebraic operations. In this paper, we propose a new method named PredLMM approximating the aforementioned LMM motivated by the concepts of genetic coalescence and Gaussian predictive process. PredLMM has substantially better computational complexity than most of the existing LMM based methods and thus, provides a fast alternative for estimating heritability in large scale cohort studies. Theoretically, we show that under a model of genetic coalescence, the limiting form of our approximation is the celebrated predictive process approximation of large Gaussian process likelihoods that has well-established accuracy standards. We illustrate our approach with extensive simulation studies and use it to estimate the heritability of multiple quantitative traits from the UK Biobank cohort.

## Author summary

In recent years, there is an increased interest of estimating heritability from genome-wide SNP data in large scale cohort studies. Here, we propose the PredLMM, a computationally rapid and memory-efficient linear mixed model for heritability estimation. The proposed approach can estimate SNP heritability on Biobank-scale datasets in a fraction of time compared to the existing mixed model based approaches. Along with the extensive simulations illustrating the precision and robustness of the PredLMM, we have also estimated heritability of several anthropometric traits from the UK Biobank cohort.

## Introduction

In the past few decades, genome-wide association studies (GWASs) have identified hundreds of single nucleotide polymorphisms (SNPs) influencing the genetic architecture of complex diseases and traits. For majority of the traits, however, the associated SNPs from a GWAS only explain a small fraction of the heritability estimated using twin and family studies. In search of this so called “missing heritability”, there were attempts to capture even infinitesimal SNP effects by taking into account genome-wide variants in a linear mixed model (LMM) framework [1–4]. This SNP-based LMM framework usually involves distantly related people, whose extent of genetic relatedness depend on their evolutionary history [5]. The total trait variance in this LMM approach is decomposed into two variance components such as the additive genetic variance and the residual variance. [6–8]. The approach requires computation and inversion of a high-dimensional genetic relationship matrix (GRM) from the genome-wide SNP data of dimensionality same as the sample size. Heritability is calculated as the ratio of the additive genetic variance to the total variance. There are softwares [1, 3, 9] which follow restricted maximum likelihood (REML) approach for estimation of the parameters and are collectively referred to as genome-based restricted maximum likelihood (GREML) methods.

In recent years, advances in genome sequencing have generated huge amount of genetic data on large scale cohort studies, such as UK Biobank [10], Precision Medicine cohort [11], Million Veterans Program [12]. These studies collect data on millions of genetic markers and numerous diseases/traits on thousands of individuals. For example, UK Biobank cohort has data on approximately 500,000 individuals, 800,000 markers and numerous traits. Therefore, it is needless to say that GREML methods need to be extremely time and memory efficient to be applicable on such magnanimous studies.

Programs such as genome-wide complex trait analysis (GCTA) [1], genome-wide efficient mixed model association (GEMMA) [9] have implemented efficient algorithms to fit the GREML approach. These programs usually follow two steps: first, compute the genetic relationship matrix (GRM) with the SNP data on the individuals and second, use the computed GRM to fit a GREML corresponding to a trait. If *N* be the number of individuals and *M* be the number of SNPs, the first step of computing the GRM, takes complexity of *O*(*MN*^2^) FLOPS (floating point operations). And, the next step i.e., fitting the REML to estimate heritability, requires inverting the GRM matrix which uses per iteration complexity of *O*(*N*^3^) FLOPS. When *N* is extremely large (say more than 100,000), this step becomes computationally intractable. It should be noted that the first step (computing the GRM) is also very demanding in terms of both computation and memory requirements (especially when *M*, *N* both are large). In large biobank-scale studies, where the interest is to estimate heritability of a large number of traits, implementing these approaches becomes computationally very demanding.

Recently, an approximate method named Bolt-REML [3, 13, 14] has been proposed that trades off small amount of accuracy in favor of greater computational speed. It follows a different path than the above methods. It does not compute the GRM but uses the SNP data directly to fit the REML by monte carlo average information REML algorithm. It has computational complexity of *O*(*MN*^1.5^) per iteration which is better than the previous methods in terms of *N*. The software is well optimized and in our analysis of UK Biobank data, it performed much better compared to the other approaches in terms of computational time. However, the complexity of Bolt-REML is not linear in *N* which makes it challenging to use for larger *N* (>300, 000). Additionally, the computational complexity also increases linearly with *M* per iteration. Thus, in a large cohort with millions of SNPs, it would be immensely intensive to use Bolt-REML for estimating heritability of all the traits one by one. On the other hand, methods like GCTA, GEMMA estimate the GRM only once (with computational complexity of *O*(*MN*^2^)) and after that, the complexity of analyzing any trait does not depend on *M*.

Alternative to the REML estimation approaches, there are other ways of estimating heritability from the LMM framework that can be computationally much faster but cost significant efficiency [15–17]. There are also methods like LDAK [18], MultiBLUP [19] which are based on more realistic assumptions than the standard LMM framework considered in the GCTA-GREML methods. They have been shown to produce more robust estimates of heritability [20, 21]. In this paper, however, we limit our focus on the GCTA-GREML model and propose a scalable alternative.

In this paper, we approximate the likelihood of the standard LMM framework to develop a rapid algorithm for estimating heritability. The approximation is motivated by the concepts of genetic coalescence [22, 23] and Gaussian predictive process models [24, 25]. Our proposed approach PredLMM exploits the structure of the GRM to ease the computationally demanding linear algebraic steps of the standard GREML algorithm, such as calculation of the determinant or inverse of a high dimensional matrix (*N* × *N*) at every iteration. It reduces per iteration computational complexity from *O*(*N*^3^) FLOPS (floating point operations) to *O*(*Nr*^2^) + *O*(*r*^3^) FLOPS where *r* is much smaller than *N*. Theoretically, we show that under a model of genetic coalescence, the limiting form of our approximation is the celebrated predictive process approximation of large Gaussian process likelihoods [24] that has well-established accuracy standards. The method does not require computing and storing the full GRM which would take up *O*(*N*^2^) storage and cost a significant amount of time. Our approach stores only a few blocks of the GRM and requires only a storage space of *O*(*Nr* + *r*^2^). We have demonstrated that the proposed approach achieves accuracy close to the GREML methods through extensive simulation studies replicating many possible realistic scenarios. We have analyzed the UK Biobank cohort data (with 286,000 British individuals and 566,000 SNPs) to estimate the heritability of *Standing Height, Weight, BMI, Systolic and Diastolic blood pressure, Hip and Waist circumference*. We have implemented PredLMM in an efficient Python module available at this link, (https://github.com/sealx017/PredLMM). It is worth pointing out that in the developed module, we allow users to incorporate SNP-based weights, such as LD-based weights proposed by Speed et. al. (2012) [18], into the GRM-estimation.

## Methods

### Genome-based restricted maximum likelihood

#### Model specification

Let **Y** denote the *N* × 1 vector of phenotype corresponding to *N* individuals, **X** denote the *N* × *p* matrix of covariates, and **W** denote the *N* × *M* matrix of mean and variance scaled genotype for the *N* individuals and *M* SNPs, i.e., *E*(*w*_*ij*_) = 0 and *Var*(*w*_*ij*_) = 1. Consider the following LMM,

$$\begin{array}{c} Y=X\beta +W\gamma +\epsilon ;\;\gamma ∼N_{N}\left(0,\sigma_{w}^{2}I\right),\;\epsilon ∼N_{N}\left(0,\sigma_{e}^{2}I\right) \end{array}$$ (1)

And, the corresponding marginal model can be written as,

$$\begin{array}{c} Y∼N_{N}\left(X\beta ,\sigma_{h}^{2}A+\sigma_{e}^{2}I\right);\;\sigma_{h}^{2}=M\sigma_{w}^{2},\;A=\frac{1}{M}WW^{⊤} \end{array}$$ (2)

where **A** is formally known as the Genetic Relationship Matrix (GRM) and **I** is the identity matrix. Heritability is calculated as $h^{2}=\sigma_{h}^{2}/\left(\sigma_{h}^{2}+\sigma_{e}^{2}\right)$.

#### Estimation approaches

To estimate the variance parameters $\sigma_{h}^{2},\sigma_{e}^{2}$ and eventually *h*^2^, different REML algorithms are generally used. The entire framework is referred to as genome-based restricted maximum likelihood (GREML) approach. There are two types of programs implementing the GREML approach: a) Exact Methods (methods that converge to the REML optimum) and b) Approximate Methods (methods that approximate the REML optimum).

*Exact Methods:* Programs such as GCTA [1], GEMMA [26] operate in two steps: first, compute the GRM, $A=\frac{1}{M}WW^{⊤}$ and second, consider the computed **A** in the marginal model from Eq 2 to estimate *h*^2^ using different REML algorithms. These REML algorithms are iterative and compute analytically exact solutions. For example, GEMMA uses a modified version of Newton-Raphson method (considers exact Hessian), GCTA uses average information (AI) method (considers approximate Hessian and hence, computationally faster). The second step involves computing the inverse and determinant of the *N* × *N* dense matrix $V=\sigma_{h}^{2}A+\sigma_{e}^{2}I$ at every iteration which takes *O*(*N*^3^) FLOPS, making these exact methods computationally intractable as *N* increases.

*Approximate Methods:* Unlike the above methods, Bolt-REML [3, 13, 14] does not compute the GRM **A**. It directly uses with the SNP data matrix **W** and follows a Monte Carlo REML approach that uses random sampling to approximate the derivatives of the log likelihood corresponding to the marginal model from Eq 2. The algorithm has computational complexity of *O*(*MN*^1.5^) per iteration which is better than the previous methods in terms of *N*. The software is well optimized and in our analysis of UK Biobank data, unlike the previous methods, it would successfully converge for moderately large *N* (*N* > 100, 000) in a reasonable amount of time. However, the per iteration computational complexity of Bolt-REML still increases linearly with *M*. Thus, in a cohort study where *M* is closer to a million (or, larger), it will become computationally much more challenging to use Bolt-REML. On the other hand, methods like GCTA, GEMMA estimate the GRM only once (with computational complexity of *O*(*MN*^2^)) and after that, the complexity of analyzing any trait does not depend on *M*.

### Sub-sample based GREML

Since, the likelihood based methods above involving the full population become increasingly computationally demanding as the population size *N* increases, an alternative would be to utilize a sub-sample based approach. Choose a random sub-sample of small size *r* from the pool of all *N* individuals and use a standard GREML based program, such as GCTA to estimate heritability (${\hat{h}}_{\text{sub}}^{2}$). Asymptotically, ${\hat{h}}_{\text{sub}}^{2}$ should be consistent but have a much higher variance than the full data based GREML estimate.

In our simulation studies and real data analysis, we assess the performance of this method for varying values of *r* and refer to it as GREML (sub).

### Proposed method

#### Asymptotic limit of the GRM

To motivate our method, we first show that under certain assumptions, as the number of SNPs *M* goes to infinity, the likelihood corresponding to the marginal model from (2) converges almost-surely to a Gaussian process (GP) likelihood. The assumptions are as follows,

1. *Assumption 1 (Correlation across individuals):* We assume that each individual *i* = 1, 2, …, *N* can be represented by a point (location) **s**_*i*_ in an abstract spatial manifold D equipped with a distance *d*. The correlation between the genotypes of individuals *i* and *i*′ at the *j*^*th*^ SNP is given by *Cov*(*w*_*ij*_, *w*_*i*′*j*_) = *C*_*j*_(**s**_*i*_, **s**_*i*′_) where *C*_*j*_ is a valid covariance function in D which decreases monotonically with increasing distance $d(s_{i},s_{i}^{\prime })$.
  This assumption is rooted in the theory of *genetic coalescence* [22, 23]. The coalescence model describes the relationships within a sample from the present individuals (sequences) back to the most recent common ancestor (MRCA) [27]. Under coalescence, the correlation between genotypes of individuals will vary inversely with the *time to coalescence*, i.e., number of ancestral generations till the *most recent common ancestor*. Hence, the MRCAs of different pairs of individuals in a sample can be assigned to nodes of a genealogical tree. Trees are equipped with a valid distance metric (shortest distance between nodes) and models for tree-structured objects commonly specify the correlation as decreasing function of the distance [28].
  Note that, the maximum likelihood estimate of *h*^2^ from (2) has been shown to be consistent in [29]. However, the theory relies on the assumption that the genotype distributions are independent across individuals (upto standardization). Formally, **w**_*i*_ ⊥ **w**_*i*′_ for any two individuals *i* ≠ *i*′ where **w**_*i*_ = *i*^*th*^ row of **W**, is the genotype vector for the *i*^*th*^ individual. Such an assumption of between-individual independence of genotype distributions is in sharp violation of the principles of coalescent theory.
  We note while coalescence model is a natural example where our assumption of latent embedding is realized, the concept is not just restricted to trees and can be compatible with more complex models of ancestry depicted by any manifold with a notion of distance.
2. *Assumption 2 (Stationarity and ergodicity across the SNPs):* We assume that the centered and scaled genotype process ${\tilde{w}}_{j}={\left(w_{1j},\ldots ,w_{Nj}\right)}^{\prime }$ is second-order stationary and ergodic for *j* = 1, 2, …. Stationarity translates to $Cov\left({\tilde{w}}_{j}\right)=Cov\left({\tilde{w}}_{j^{\prime }}\right)=C$ for all *j*, *j*′ implying that the covariance functions *C*_*j*_ = *C* for all *j* = 1, 2, … Ergodicity implies that as the number of SNPs grows, we have
  $$\begin{array}{c} \underset{M\to \infty }{\text{lim}}A=\underset{M\to \infty }{\text{lim}}\frac{1}{M}\sum_{j=1}^{M}{\tilde{w}}_{j}{\tilde{w}}_{j}^{⊤}\to C=Cov\left({\tilde{w}}_{1}\right) \end{array}$$ (3)
  The simplest setting where this assumption is satisfied is when the scaled and centered genotype processes ${\left\{{\tilde{w}}_{j}\right\}}_{j=1,2,\ldots }$ are assumed to be iid. Assumption of iid genotypes is common in theoretical studies of the heritability estimation [29] but independence is only sufficient and not necessary for us. More realistic scenarios like presence of linkage disequilibrium (LD) that effectuates correlation across genotypes can also be accommodated as long as the ergodicity is ensured. As shown in [30], the pairwise LD among loci in a homogeneous population decreases exponentially as a function of the genetic distance, which validates the feasibility of our assumption. Correlation structures arising from absolutely regular-mixing processes [31] like autoregressive (*AR*(*p*)), moving average (*MA*(*q*)) or *ARMA*(*p*, *q*) [32] will satisfy the strong law of convergence in Eq (3) [33].

Under Assumption 2, we have the following assertion on the limit of the marginal LMM likelihood from (2),

$$\begin{array}{cc} & \underset{M\to \infty }{\text{lim}}N_{N}\left(Y\;|\;X\beta ,\sigma_{h}^{2}A+\sigma_{e}^{2}I\right)=N_{N}\left(Y\;|\;X\beta ,\sigma_{h}^{2}C+\sigma_{e}^{2}I\right)\; \end{array}$$ (4)

where *N*_*N*_(**Y**|***μ***, **Σ**) denotes the *N*-variate normal likelihood for a realization **Y** with mean ***μ*** and variance **Σ**. Thus the marginal GREML likelihood converges to the likelihood of a Gaussian process (on D) with mean 0 and covariance function *C* observed at the *N* latent locations **s**_1_, **s**_2_, …, **s**_*N*_. It is expected that estimation of heritability using the limiting likelihood (4) will be similar to that from the exact likelihood (2) as the number of SNPs *M* is usually very large.

#### PredLMM

Just switching to the limiting likelihood (4) does not ease any of the computational burden as GP likelihoods also require *O*(*N*^3^) FLOPS. However, over the last two decades a series of increasingly sophisticated algorithms have been proposed for fast approximate GP likelihoods (see [34], for a recent review).

Our approach uses predictive process (PP) [24, 25] which results in the low-rank plus diagonal approximation of the dense matrix **C**. Let $S=\{s_{1},s_{2},\ldots ,s_{N}\}$ denote the set of *N* latent locations, and $S^{*}=\left\{s_{1},s_{2},\ldots ,s_{r}\right\}$ denote a set of *r* ≪ *N* locations in D referred to as the *knots*. Also, for two sets *A* and *B* in D let **C**_*A*,*B*_ denote the |*A*| × |*B*| matrix (*C*(**s**_*i*_, **s**_*i*′_))_*i*∈*A*,*i*′∈*B*_. The predictive process approximation of **C** is given by

$$\begin{array}{c} {\tilde{C}}_{PP}=C_{S,S^{*}}C_{S^{*},S^{*}}^{-1}C_{S^{*},S}+diag\left(C-C_{S,S^{*}}C_{S^{*},S^{*}}^{-1}C_{S^{*},S}\right). \end{array}$$ (5)

The first term is a low-rank factorization as the number of knots is much less than the sample size. [24] showed that this low-rank term is the optimal (in terms of reverse Kullback Leibler divergence) low-rank approximation of **C** using the knots S*. [25] proposed adding the diagonal matrix (second term) to eliminate a positive bias on the diagonal entries. For moderate choices of *r* ≪ *N*, inference from the predictive process likelihood provides an excellent approximation to that from the full GP likelihood. Computationally, predictive process only requires *O*(*Nr*^2^ + *r*^3^) FLOPS and as *r* ≪ *N*, the approximation results in massive gains in run times. Consequently, predictive processes is one of the most popular approximations of the full GP likelihood and is widely adopted in many spatial applications.

In our setting, direct usage of predictive process likelihood is not recommended for two reasons. First, the locations **s**_*i*_ are unknown to us. Hence, **C**_*PP*_ can only be calculated using approximate locations like a vector of the top few PC scores. The impact of such choices of locations is less clear. Second, covariance functions usually involve additional spatial parameters ***θ***, thereby increasing the number of unknown parameters to be estimated.

Instead, we consider the following strategy. We choose $S^{*}$ to be a subset of S, and define I to be the subset of B={1,2,…,N} containing the indices corresponding to $S^{*}$. We can decompose the GRM **A** as,

$$\begin{array}{cc} & A=A_{B,B}=A_{B,I}A_{I,I}^{-1}A_{I,B}+\left(A_{B,B}-A_{B,I}A_{I,I}^{-1}A_{I,B}\right) \end{array}$$

The decomposition is inspired by the concept of conditional variance [35]. The first term $A_{B,I}A_{I,I}^{-1}A_{I,B}$ on the right is the low-rank part of the full GRM **A** that is explained by the information about the subset of individuals I, while the second term $A_{B,B}-A_{B,I}A_{I,I}^{-1}A_{I,B}$ is the residual GRM of the individuals in the subset $B\cap I^{c}$ that is not explained by the individuals in the subset I. Replacing the term on the right with its diagonal, we then have a direct low-rank plus diagonal approximation of **A** as

$$\begin{array}{c} {\tilde{A}}_{PP}=A_{B,I}A_{I,I}^{-1}A_{I,B}+diag\left(A_{B,B}-A_{B,I}A_{I,I}^{-1}A_{I,B}\right) \end{array}$$ (6)

We propose using the likelihood $N_{N}\left(Y\;|\;X\beta ,\sigma_{h}^{2}{\tilde{A}}_{PP}+\sigma_{e}^{2}I\right)$ for heritability estimation. It is clear that **A** and ${\tilde{A}}_{PP}$ agree on the diagonals, and on the sub-matrix corresponding to the knots I. Also, ${\text{lim}}_{M\to \infty }{\tilde{A}}_{PP}={\tilde{C}}_{PP}$ (since the individual terms of ${\tilde{A}}_{PP}$ follow: ${\text{lim}}_{M\to \infty }A_{BI}=C_{S,S^{*}}$, ${\text{lim}}_{M\to \infty }A_{IB}=C_{S^{*},S}$ and ${\text{lim}}_{M\to \infty }A_{II}=C_{S^{*},S^{*}}$ using Eq 3). Hence, using triangular inequality, we can write

$$\begin{array}{c} ∥A-{\tilde{A}}_{PP}∥\leq ∥A-C∥+∥C-{\tilde{C}}_{PP}∥+∥{\tilde{C}}_{PP}-{\tilde{A}}_{PP}∥, \end{array}$$

where ||.|| is the Frobenius norm [36]. Under assumption 2, the first and third terms vanish as *M* → ∞, while for a well chosen set of knots S*, the predictive process approximation ${\tilde{C}}_{PP}$ is close to **C** (since **C** is a decreasing function of the distance as postulated in Assumption 1). Hence the middle term will also be small. This justifies why for large *M*, ${\tilde{A}}_{PP}$ is expected to be close to **A**.

In our empirical studies detailed later, the predictive process approximation consistently and substantially outperforms the subsample-based method when both uses the same set of knots (sub-sample) I. We offer some insight into this. The first term of ${\tilde{A}}_{PP}$ in Eq 6 is,

$$\begin{array}{c} A_{B,I}A_{I,I}^{-1}A_{I,B}=(\begin{array}{cc} A_{I,I} & A_{I,I^{c}}\\ A_{I^{c},I} & A_{I^{c},I}A_{I,I}^{-1}A_{I,I^{c}} \end{array}). \end{array}$$ (7)

As mentioned before, this low-rank matrix is the best estimate of **A** based on the genetic information only from the individuals in subset I and their genetic correlation with the individuals in subset $I^{c}$. If using the sub-sampling based approach with the same sub-sample I, one would only use the sub-matrix $A_{I,I}$ to estimate *h*^2^. This thus ignores the genetic correlation of these sub-sampled inviduals with those not sub-sampled (quantified as $A_{I,I^{c}}$), and is thus sub-optimal to the predictive process approach which leverages this genetic relationship among individuals while remaining computationally scalable.

#### Computational gains

Evaluation of our PredLMM likelihood $N_{N}\left(Y\;|\;X\beta ,\sigma_{h}^{2}{\tilde{A}}_{PP}+\sigma_{e}^{2}I\right)$, does not require computing or storing the entire *N* × *N* GRM matrix **A** and can be calculated only using the *N* × *r* tall thin sub-matrix $A_{B,I}$, the small *r* × *r* square matrix $A_{I,I}$, and diagonal elements of **A**. This reduces memory requirements from *O*(*N*^2^) to *O*(*Nr* + *r*^2^)—a substantial gain for biobank-scale studies with large *N* as *r* ≪ *N*.

Subsequently, the nice low-rank plus diagonal structure of ${\tilde{A}}_{PP}$ facilitates fast evaluation of the likelihood. Inverse of $\sigma_{h}^{2}{\tilde{A}}_{PP}+\sigma_{e}^{2}I$ becomes feasible and significantly rapid using the Woodbury matrix identity [37], while the matrix determinant lemma [38] is leveraged for scalable computation of the determinant. Both the steps involve *O*(*Nr*^2^ + *r*^3^) FLOPS, as *r* ≪ *N*, the computation is thus becomes linear in *N*—a drastic reduction from the *O*(*N*^3^) FLOPS required for evaluating the true likelihood.

#### Choice of knots design and number

In traditional applications of Gaussian processes in spatial statistics, the domain D is known and the locations **s**_*i*_ are observed. Hence, the knots need not coincide with the data locations. Recommended choices for the knot-set include space-filling designs and lattices [24]. In our case, the locations are artificial constructs to motivate our direct approximation. Hence, restricting the knot set to be a sub-sample of these hypothetical data locations is necessary to ensure that the direct approximation ${\tilde{A}}_{PP}$ can be calculated using sub-matrices of **A**. However, our practice has precedence even in conventional spatial settings. Using some of the data locations has been shown to improve performance of predictive process [24], while related approaches like splines and other basis function expansions also commonly use data locations as knots. We used random sub-samples of the individuals as knots in the simulation studies discussed in Verification and comparison Section and it demonstrated considerable robustness to the choice of sub-sample. However, in the real data analysis, the performance seemed to improve with a more careful knot-selection. We pre-compute the sum of the entries of each row of the true GRM **A**. Next, we ordered the set of indices, B based on high to low values of row-sum and then selected first *r* individuals as the set of knots. Note that a large value of row-sum indicates that the particular individual shares significant genetic relationship with one or more other individuals and would be a more fitting choice as a knot than an individual sharing little to no correlation with the rest of the individuals. The reasoning becomes more apprehensible if we look at Eq 7. The only off-diagonal elements of ${\tilde{A}}_{PP}$ that do not exactly match those of the true GRM **A**, are the ones corresponding to the sub-matrix $A_{I^{c},I}A_{I,I}^{-1}A_{I,I^{c}}$. If we choose a set of knots I where the individuals not do not share high genetic relationship among themselves or with others ($I^{c}$), $A_{I,I}$ will be close to an identity matrix and $A_{I,I^{c}}$ close to a matrix with all 0’s. It will further cause the off-diagonal elements of $A_{I^{c},I}A_{I,I}^{-1}A_{I,I^{c}}$ to be mostly 0 and far from their true values. In short, for better prediction of the genetic relationship values between the individuals of the set $I^{c}$, choosing an informative set of knots I is important.

Choice of the the number of sub-samples *r* to be used for PredLMM is more nuanced. Performance of predictive process is generally more sensitive to the number than the design of the knots [24, 39]. Increasing *r* improves the quality of the approximation, with ${\tilde{A}}_{PP}$ exactly equalling **A** when *r* = *N* and I=B. However, as the computation is cubic in *r*, use of a very large *r* would defeat the purpose of the approximation. Parallel computing resources, if available, can be heavily deployed for this step.

#### Asymptotic variance of the estimator

We have derived the expression of the asymptotic variance (standard error) of the PredLMM estimator. Since it is extremely time consuming to perform the matrix multiplications needed for the exact computation of the variance expression, we make some reasonable approximations. The details of the derivation can be found in S1 Appendix.

## Verification and comparison

### Simulation Study 1: Simulation under coalescent model

**Fig 1:**
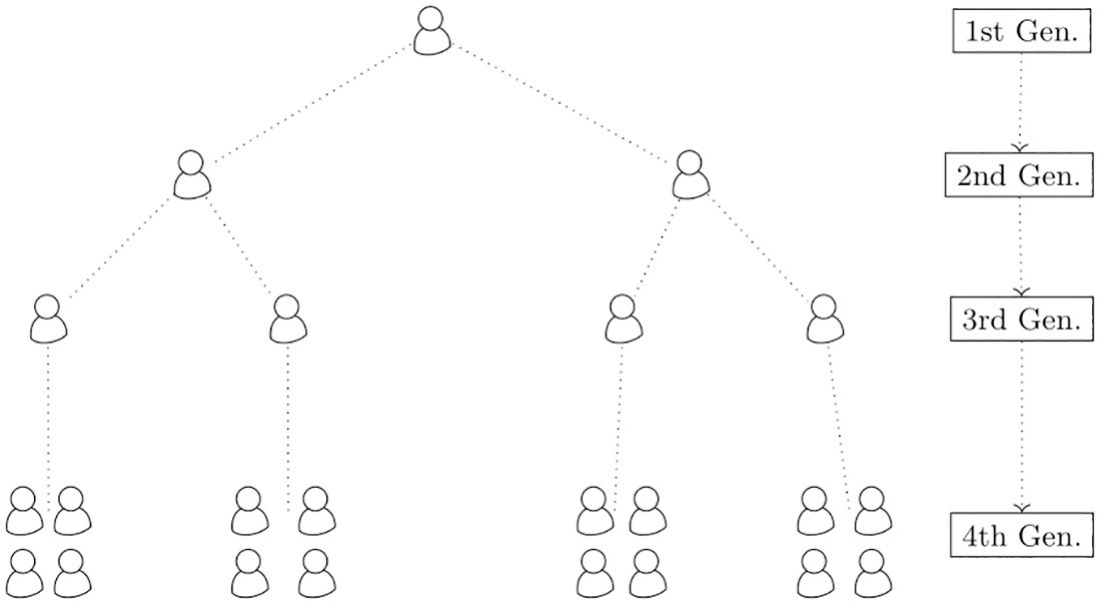
The figure shows a model coalescence with four generations. Each of the four blocks of people in the fourth generation share one of the individuals from the third generation as common ancestor. Similarly, the four people in the third generation have originated from the two in the second generation. And, finally those two people have originated from a common ancestor in the first generation.

The following simulation study replicated a scenario where Assumption 1 from Section Asymptotic limit of the GRM approximately held i.e., every individual originated from a common ancestor and individuals in the same sub-population shared a more recent ancestor than the individuals in different sub-populations. Such an evolutionary tree-like structure with four generations has been depicted in Fig 1, based on which we generated the population.

The generation procedure was as follows. For each SNP *j* (*j* = 1, …, *M*), the allele frequency $p_{j}^{\left(0\right)}$ in the first generation was drawn from a uniform distribution on [0.1, 0.9]. In the second generation, allele frequencies of two different individuals: $p_{1j}^{\left(1\right)},p_{2j}^{\left(1\right)}$ were independently simulated from a beta distribution with parameters $p_{j}^{\left(0\right)}\left(1-\theta \right)/\theta$ and $\left(1-p_{j}^{\left(0\right)}\right)\left(1-\theta \right)/\theta ,\theta =0.05$. This model is commonly known as Balding-Nichols model [40, 41]. In the third generation, allele frequencies of two individuals: $p_{1j}^{\left(2\right)},p_{2j}^{\left(2\right)}$ were independently drawn from a beta distribution with parameters $p_{1j}^{\left(1\right)}\left(1-\theta \right)/\theta$ and $\left(1-p_{1j}^{\left(1\right)}\right)(1-\theta /\theta$ and allele frequencies of other two individuals: $p_{3j}^{\left(2\right)},p_{4j}^{\left(2\right)}$ were independently drawn from a beta distribution with parameters $p_{2j}^{\left(1\right)}\left(1-\theta \right)/\theta$ and $\left(1-p_{2j}^{\left(1\right)}\right)\left(1-\theta \right)/\theta$. Finally, in the fourth generation, the allele frequency of *j*-th SNP of the *i*-th individual from the *k*-th sub-population (*k* = 1, …, 4): *p*_*ijk*_ was simulated from a beta distribution with parameters $p_{kj}^{\left(2\right)}\left(1-\theta \right)/\theta$ and $\left(1-p_{kj}^{\left(2\right)}\right)\left(1-\theta \right)/\theta$. We kept the size of each of the four sub-populations at *N*/4 resulting in a total population of size *N*. Next, we simulated the SNP genotype: *w*_*ijk*_ from a binomial distribution: *Bin*(2, *p*_*ijk*_) assuming Hardy-Weinberg equilibrium. Once the genotypes of *M* SNPs for *N* individuals are simulated, we randomly selected *m*_*causal*_ causal SNPs (out of *M*) to create a *N* × *m*_*causal*_ causal SNP genotype matrix denoted by **W**^*causal*^. Fixed effect of *m*-th causal SNP: *u*_*m*_ was simulated from *N*(0, *h*^2^/*m*_*causal*_), and the residual effect *e* was simulated from *N*_*N*_(**0**, (1/*h*^2^ − 1)**I**_*N*_). Finally, the *N*-dimensional phenotype vector (**Y**) was generated as, $Y=\sum_{m=1}^{m_{causal}}W_{m}^{causal}u_{m}+e$, where $W_{m}^{causal}$ was the *m*-th column of **W**^*causal*^.

**Fig 2:**
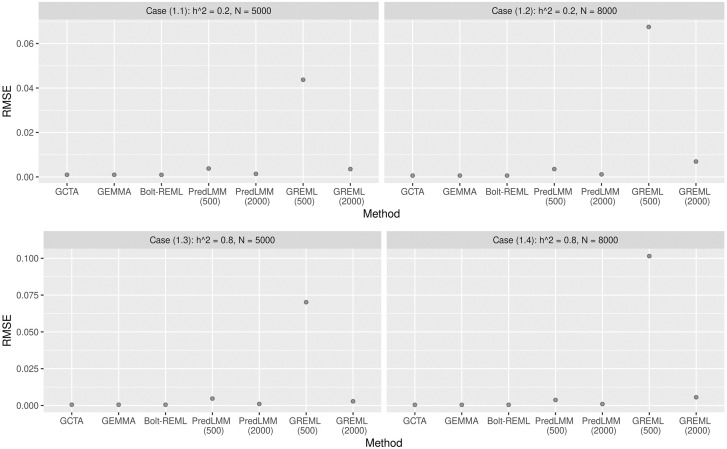
The figure shows the empirical RMSE of different methods from Simulation Study 1. Each of the four sub-plots corresponds to four different cases. For every case, 100 replications were considered. GREML (sub) had very high RMSE compared to PredLMM and the latter had RMSE close to the full GREML based methods.

We considered two different values of the true heritability: *h*^2^ (low and high) and two different combinations of the number of individuals *N* and the number of SNPs *M*. We considered case (1.1): *h*^2^ = 0.2, *N* = 5000, *M* = 8000, case (1.2): *h*^2^ = 0.2, *N* = 8000, *M* = 13000, case (1.3): *h*^2^ = 0.8, *N* = 5000, *M* = 8000 and case (1.4): *h*^2^ = 0.8, *N* = 8000, *M* = 13000 to study the influence of *M* and *N* on heritability estimation. In this simulation study and also in the subsequent ones we considered 100 replications. Fig 2 shows the empirical root mean-squared error (RMSE) of different methods. RMSE is defined as the square root of the sum of the squared bias and the variance of an estimator. Thus, a comparison of empirical RMSE assesses the quality of the estimators both in terms of their variation and their bias [42]. We considered several full likelihood based GREML methods discussed earlier: GCTA, GEMMA and Bolt-REML for comparison with PredLMM. Since all of these methods maximize the full likelihood corresponding to the marginal model in (2), their estimates were expected to be precise and close to each other. Consistent with the expectation, the methods showed very close empirical RMSE in Fig 2. GREML (500) and GREML (2000) referred to the sub-sample based GREML with sub-sample sizes of 500 and 2000 respectively. PredLMM (500) and PredLMM (2000) referred to fitting PredLMM with knot-sizes (*r*) 500 and 2000 respectively. We noticed that GREML (500) had the largest empirical RMSE in all the cases with the largest being in case (1.4). PredLMM (500) showed RMSE values close to GREML (2000), whereas PredLMM (2000) achieved RMSE close to the full GREML based methods, such as GCTA, GEMMA and Bolt-REML. Therefore, we could conclude that when the genetic data were simulated using the Balding-Nichols model, the quality of the PredLMM estimator would be much superior compared to the sub-sample based GREML and even close to the full GREML based methods for a moderately large knot-size. Refer to S2 Fig for the box-plots of the estimates to visualize the empirical bias and precision of the estimates. We noticed that PredLMM estimates were unbiased and had very little spread i.e., much better precision compared to GREML (sub).

### Simulation Study 2: Simulation using UK Biobank data

To replicate more realistic scenarios, we next considered simulations using the UK Biobank cohort data [10]. UK Biobank is a large long-term biobank study in the United Kingdom which is investigating the respective contributions of genetic predisposition and environmental exposure to the development of various diseases. We had access to 784,256 markers and multiple phenotypes on 502,628 individuals. The population is predominantly British (442,687) with a few other ethnicities such as Irish (13,213), Other White (16,340), Asian (9839), and Black (8038). There is clear genetic clustering in the UK Biobank population that has been explored in [43].

**Fig 3:**
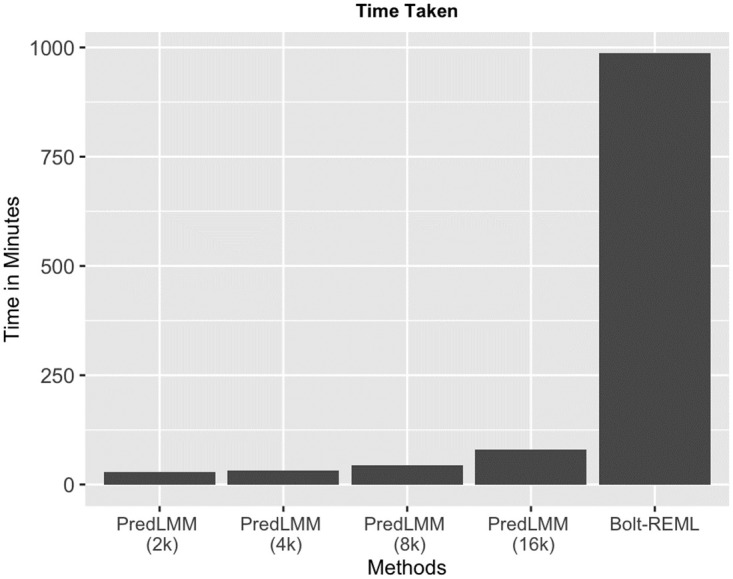
The figure shows the time taken by PredLMM with different knot-sizes such as, 2000, 4000, 8000 and 16,000 and by Bolt-REML for a single simulation with 100,000 individuals and 566,000 SNPs (from Simulation Study 2).

After standard quality control steps as advised in [44] (removing SNPs with MAF less than 0.01 and missingness over 10%, removing individuals with high missing genotype rate), we had approximately 320,000 individuals and 566,000 SNPs. Since, conducting simulation studies with the entire dataset would be very computationally expensive, we created a mixture sub-population of lesser size, 157,000 people (120,000 British and 37,000 from other ancestries such as Asian, Black, Irish, and Indians). Majority of the full GREML-based methods such as GCTA, GEMMA were computationally infeasible for such a large number of individuals. Bolt-REML was the only full GREML-based method that would still be viable in this context. But, as we saw from Fig 3 that even for a single simulation with 100,000 individuals, Bolt-REML took approximately 1000 minutes to run (more details regarding the time comparison can be found in Time comparison Section). Therefore, we only compared PredLMM with GREML (sub) in the subsequent simulations. Keeping the genetic heterogeneity in mind, we looked into two different simulations using the genetic data from the UK Biobank study, (2.1), one with homogeneous sub-populations and (2.2), another one with heterogeneous sub-populations. For each replication, in study (2.1), we randomly selected 100,000 people with only British ancestry from the sub-population of 157,000 people, and in study (2.2), we randomly selected 100,000 people not restricting their ancestry from the same sub-population. We considered three different true values of heritability (low to high): (a) *h*^2^ = 0.2, (b) *h*^2^ = 0.4, and (c) *h*^2^ = 0.6. Next, we simulated the trait as **Y**_100,000_ ∼ *N*_100,000_(**0**, 250*h*^2^ **A**_100,000_ + 250(1 − *h*^2^)**I**_100,000_) where **A**_100,000_ was the corresponding GRM. We compared PredLMM with GREML (sub) for four different sub-sample (knot) sizes, *r* = 2000, 4000, 8000, and 16, 000.

**Fig 4:**
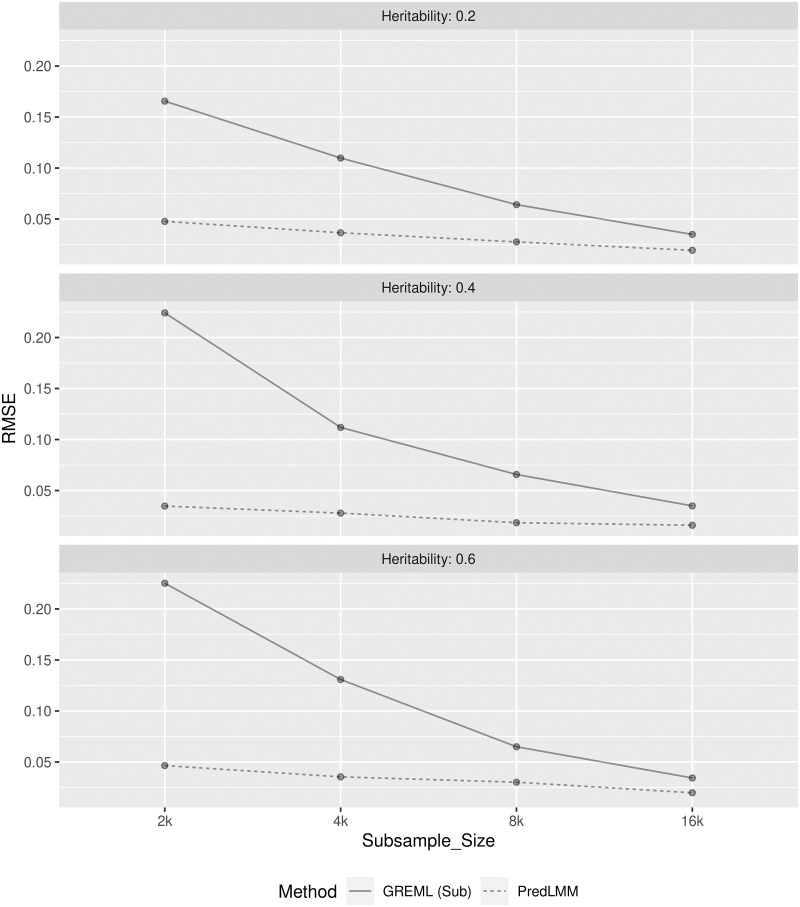
The figure shows the empirical RMSE of GREML (sub) and PredLMM for four different sub-sample (knot) sizes: 2000, 4000, 8000, 16000 in cases (a), (b) and (c) from Simulation Study (2.1). For every case, 100 replications were considered. GREML (sub) had very high RMSE for smaller knot-sizes and it became increasingly closer to PredLMM as the knot-size increased.

**Fig 5:**
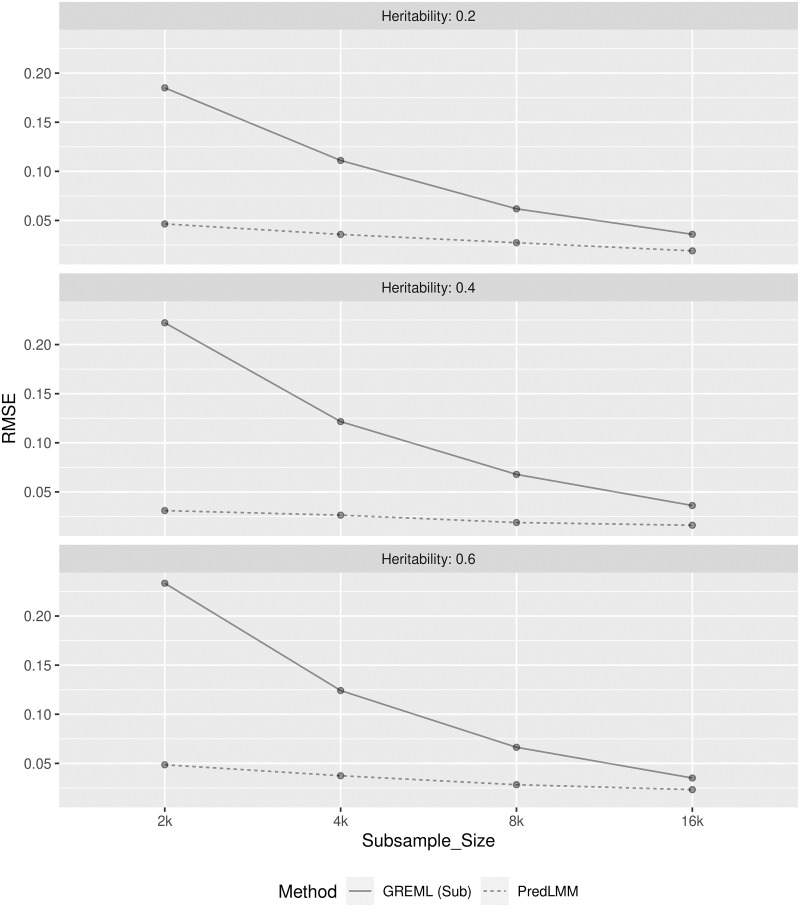
The figure shows the empirical RMSE of GREML (sub) and PredLMM for four different sub-sample sizes: 2000, 4000, 8000, 16000 in cases (a), (b) and (c) from Simulation Study (2.2). For every case, 100 replications were considered. GREML (sub) had very high RMSE for smaller knot-sizes and it became increasingly closer to PredLMM as the knot-size increased.

Empirical RMSE comparison of the estimators for study (2.1) and (2.2) are respectively shown in Figs 4 and 5. In both the studies, GREML (sub) showed much larger RMSE compared to PredLMM especially for smaller sub-sample sizes like 2000 and 4000. The gap between the RMSE of the estimators kept narrowing as the sub-sample size increased. However, even for the largest sub-sample size, 16000 the gap remained prominent demonstrating PredLMM’s superior quality. To visualize the empirical bias and precision of the estimates, refer to the box-plots from S3 and S4 Figs. We noticed that the spread of the estimates were the largest for GREML (sub). In both the studies, PredLMM showed slight downward bias when the true heritability was high (case (c)) and slight upward bias when the true heritability was low (case (a)). For moderate value of heritability (case (b)), the bias was negligible even for the smallest knot-size, 2000.

### Time comparison

The huge time advantage of PredLMM has already been illustrated in Fig 2. Here, we present a few more tables in support of that and specify all the technical details. We ran all the methods on a HP Linux cluster with nodes that use 24 many Haswell E5–2680v3 processor cores and has RAM of 248 GB. We have listed in Tables 1 and 2 the time taken by different methods for Simulation Study 1 and for Simulation Study (2.2) respectively. From Table 1, we noticed that the methods like GCTA and Bolt-REML took similar amount of time, whereas PredLMM with 500 knots took around 40% of that. PredLMM with 2000 knots takes time similar to Bolt-REML. The time advantage was more prominent in Table 2 (this comparison is also shown in Fig 3).

**Table 1:** Time comparison of different methods in seconds for Simulation Study 1 with 5k (8k SNPs) and 8k (13k SNPs) individuals.

	GCTA	GEMMA	Bolt-REML	GREML (500)	GREML (2000)	PredLMM (500)	PredLMM (2000)
5k	15.5	351.07	13.25	3.41	6.7	5.398	16.77
8k	33.5	1293.44	27.87	5.7	3.3	13.67	28.46

**Table 2:** Time comparison (in minutes) of PredLMM for varying different knot (sub-sample) sizes with Bolt-REML for Simulation Study (2.2) with 100k individuals.

Knot size	PredLMM				Bolt-REML
	2000	4000	8000	16000	
Time	28.33	31.41	44.17	80	986.4

According to Table 2, PredLMM took just a fraction (around 8%) of time compared to Bolt-REML even if we choose a large knot size of 16,000. PredLMM takes very similar amount of time for knot sizes 2000 and 4000. We noticed a significant leap in the run-time from knot size of 8000 to knot size of 16,000. Recall that the per iteration computational complexity of PredLMM is *O*(*Nr*^2^ + *r*^3^) i.e., the complexity is cubic with respect to the knot size *r* which justifies the leap. One may argue that it would be wise to use just 8000 knots since it can yield a reasonable estimate in a very reasonable time. We should also mention that we used a pre-computed GRM (using GCTA) in all our analyses (we computed the GRM for the entire population and used its sub-matrices as necessary in Simulation Study 2). Computing the GRM is an arduous task that can take multiple hours depending upon the number of SNPs and the number of individuals. It has computational complexity of *O*(*MN*^2^). But, it is usually less concerning since the computation needs to be performed only once and the computed GRM then can be used in multiple analyses. Bolt-REML does not use a pre-computed GRM and uses the genetic data every time for each of the traits which makes it very time consuming to perform a heritability analysis with multiple traits.

## Applications

**Fig 6:**
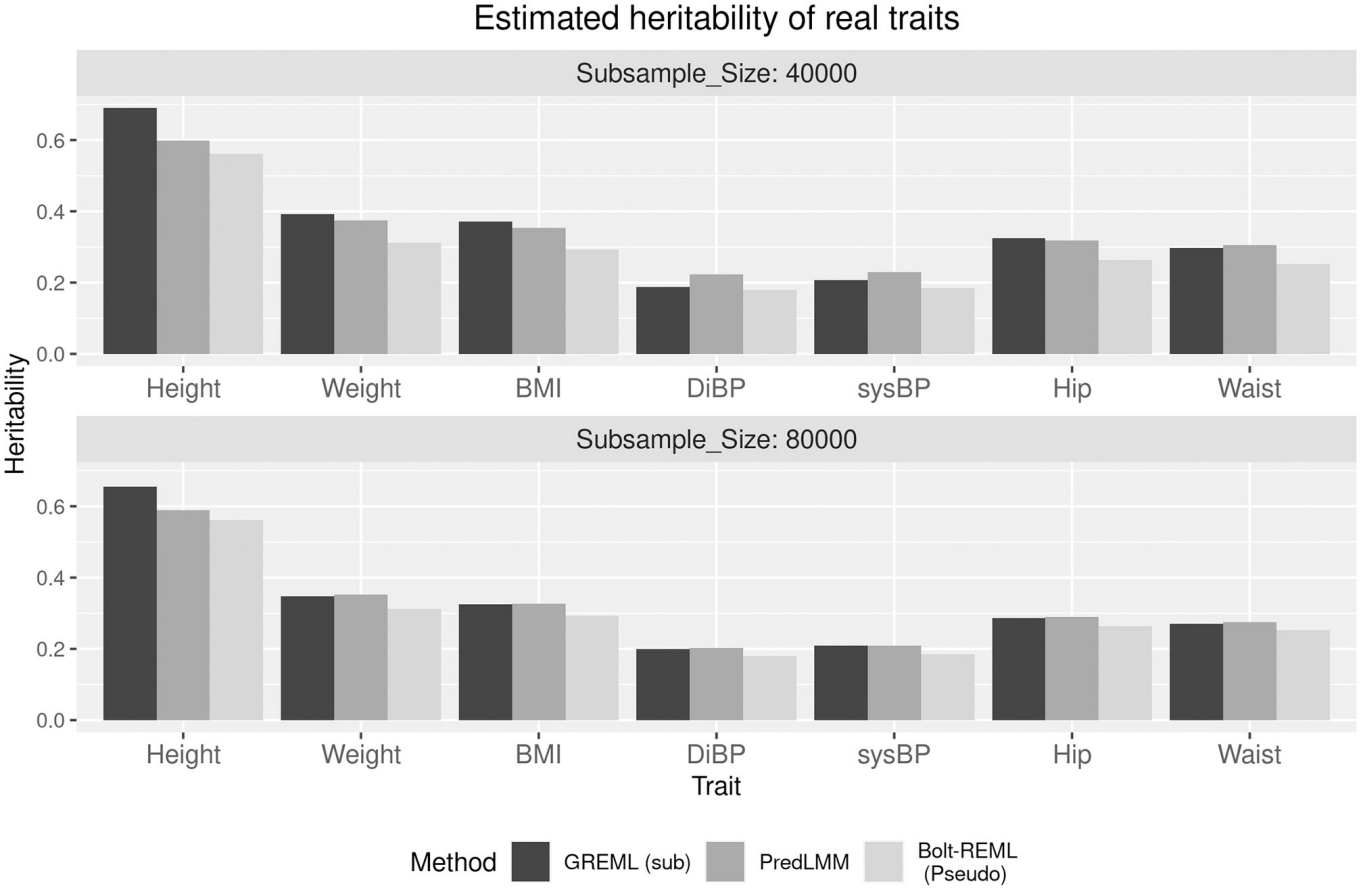
The figure shows the bar-plot of the heritability estimates by PredLMM and GREML (sub) with two sub-sample (knot) sizes and by Bolt-REML (pseudo) for seven different real traits.

We estimated heritability of a number of quantitative traits: *Standing Height, Weight, BMI, Diastolic and Systolic blood pressure, Hip and Waist circumference* using the British population of size 286,000 and 566,000 SNPs [45]. We took into account the fixed effects of covariates, such as sex, age, squared age, and the top 10 genetic principal components. We used the row-sum based knot selection technique described in Proposed method Section to select knot-sets (sub-samples) of sizes, 40,000 and 80,000 using which we ran both GREML (sub) and PredLMM Since, running the full version of Bolt-REML would take an exorbitant amount of time, we computed the approximate “pseudo-heritability” option in Bolt-REML [3, 14]. The results are displayed in the form a bar-plot in Fig 6.

The heritability estimates from all the methods generally agreed with the findings from other studies based on the UK Biobank cohort [45–48]. We noticed that PredLMM estimates were closer to Bolt-REML (pseudo) than GREML (sub) for majority of the traits. Assuming the Bolt-REML (pseudo) estimates to be the closest to the truth in this particular dataset, there is a trend of slight over-estimation with PredLMM estimates. We noticed something similar in the Simulation Study (2.1) and (2.2) for smaller heritability values. However, it is also to be kept in mind that the real traits we considered here, were all highly correlated among themselves (except Height), with some of the pair-wise correlations being more than 0.8. Therefore, it is likely that if there is an issue of over-estimation with one trait, it will be translated to other traits as well. Also, PredLMM relies on the ability to predict the genetic relationship between unselected individuals. The homogeneous British population we worked with mostly shared very small genetic relatedness, meaning that the true GRM **A** had a very small proportion of elements significantly non-zero and even those were very small in magnitude. In a dataset with more genetic relatedness, PredLMM would arguably achieve adequate accuracy even with a small knot-size.

## Discussion

Genome-based restricted maximum likelihood (GREML) approaches for estimating heritability have become widely popular with the availability of large scale cohort studies. However, majority of the existing approaches such as GEMMA, GCTA, Bolt-REML implementing GREML, either become computationally very demanding or even intractable when the number of individuals (*N*) is too large. In this paper, we have developed a rapid algorithm for estimating heritability in large scale cohort studies. Our proposed approach PredLMM approximates the likelihood of a GREML approach. The approximation is achieved by unifying the concepts of genetic coalescence and Gaussian Predictive Process models. The algorithm reduces the usual per iteration computational complexity from *O*(*N*^3^) to *O*(*Nr*^2^ + *r*^3^) where *r* (knot size) is much smaller than *N*.

In different simulation studies, we have compared the empirical root mean-squared error (RMSE) of PredLMM for different knot-sizes with existing methods. From the Simulation Study 1, we have seen that under the presence of genetic pattern (a tree like structure) among the individuals, PredLMM yields highly robust estimate of heritability even with a small knot size (*r*). To replicate more realistic scenarios, next we have performed simulation studies using the real genetic data from the UK Biobank cohort study. We have checked the performance of PredLMM in two cases, a highly homogeneous sub-population and a heterogeneous sub-population (see Simulation Study 2) for a varied range of heritability values. We have observed that even with a very small knot size (say 4% or 8% of the entire population size), PredLMM can produce an extremely precise estimate of heritability at a fraction of time taken by existing softwares like Bolt-REML. Finally, we have estimated the heritability of a number of quantitative traits like *Standing Height, Weight, BMI, Diastolic and Systolic blood pressure, Hip and Waist circumference* using the entirety of the British population from UK Biobank data. For all the traits, estimates by PredLMM for varying knot-sizes come out to be very close and also, very similar to other methods like Bolt-REML (Pseudo).

Our next goal would be to analyze behavioral traits like *Alcohol Consumption, CPD (cigarettes smoked per day)* etc. from the UK Biobank data. It would be slightly more challenging since those traits often tend to be skewed and deviate from the usual normality assumption. A very efficient module implementing PredLMM in Python is available here, https://github.com/sealx017/PredLMM. Recall that, PredLMM does not use the full GRM but only a few particular blocks of it. In the module we provide a function for computing the GRM-blocks necessary for fitting the PredLMM algorithm.

## Supporting information

S1 FigPictorial formulation of ${\tilde{\text{A}}}_{PP}$.We look at the full GRM **A** and its blocks that are used in computing ${\tilde{A}}_{PP}$. For sake of simplicity in representation, we assume that first *r* of the total of *N* individuals are in the set of knots I.(TIF)

S2 Fig Comparison of PredLMM with GREML (sub) in Simulation Study (1). Box-plots of the estimates are shown for varying sub-sample sizes (knot-sizes) in four different cases.
(TIF)

S3 Fig Comparison of PredLMM with GREML (sub) in Simulation Study (2.1). Box-plots of the estimates are shown for varying sub-sample sizes (knot-sizes) in three different cases.
(TIF)

S4 Fig Comparison of PredLMM with GREML (sub) in Simulation Study (2.2). Box-plots of the estimates are shown for varying sub-sample sizes (knot-sizes) in three different cases.
(TIF)

S1 Appendix Derivation of the asymptotic variance and computational details. We derive an approximate estimate of the asymptotic variance of the PredLMM estimator. Using the proposed variance formula, we present two tables that list coverage probability of PredLMM under different simulation setups. We also provide the details on the efficient matrix operations that PredLMM makes use of.
(PDF)

## References

[1] Yang J, Lee SH, Goddard ME, Visscher PM. GCTA: a tool for genome-wide complex trait analysis. The American Journal of Human Genetics. 2011;88(1):76–82. doi: 10.1016/j.ajhg.2010.11.011 21167468 PMC3014363

[2] Lippert C, Listgarten J, Liu Y, Kadie CM, Davidson RI, Heckerman D. FaST linear mixed models for genome-wide association studies. Nature methods. 2011;8(10):833. doi: 10.1038/nmeth.1681 21892150

[3] Loh PR, Tucker G, Bulik-Sullivan BK, Vilhjalmsson BJ, Finucane HK, Salem RM, et al. Efficient Bayesian mixed-model analysis increases association power in large cohorts. Nature genetics. 2015;47(3):284. doi: 10.1038/ng.3190 25642633 PMC4342297

[4] Chen H, Wang C, Conomos MP, Stilp AM, Li Z, Sofer T, et al. Control for population structure and relatedness for binary traits in genetic association studies via logistic mixed models. The American Journal of Human Genetics. 2016;98(4):653–666. doi: 10.1016/j.ajhg.2016.02.012 27018471 PMC4833218

[5] Weir BS, Anderson AD, Hepler AB. Genetic relatedness analysis: modern data and new challenges. Nature Reviews Genetics. 2006;7(10):771–780. doi: 10.1038/nrg1960 16983373

[6] Neale MC, Cardon LR, et al. Methodology for genetic studies of twins and families. STATISTICS IN MEDICINE. 1994;13:199–199.

[7] Rabe-Hesketh S, Skrondal A, Gjessing HK. Biometrical modeling of twin and family data using standard mixed model software. Biometrics. 2008;64(1):280–288. doi: 10.1111/j.1541-0420.2007.00803.x 17484777

[8] Seal S, Boatman JA, McGue M, Basu S. Modeling the Dependence Structure in Genome Wide Association Studies of Binary Phenotypes in Family Data. Behavior genetics. 2020;50(6):423–439. doi: 10.1007/s10519-020-10010-2 32804302 PMC7581561

[9] Zhou X, Stephens M. Genome-wide efficient mixed-model analysis for association studies. Nature genetics. 2012;44(7):821. doi: 10.1038/ng.2310 22706312 PMC3386377

[10] Allen NE, Sudlow C, Peakman T, Collins R, et al. UK biobank data: come and get it; 2014. 10.1126/scitranslmed.3008601 24553384

[11] Khoury MJ, Evans JP. A public health perspective on a national precision medicine cohort: balancing long-term knowledge generation with early health benefit. Jama. 2015;313(21):2117–2118. doi: 10.1001/jama.2015.3382 26034952 PMC4685667

[12] Gaziano JM, Concato J, Brophy M, Fiore L, Pyarajan S, Breeling J, et al. Million Veteran Program: a mega-biobank to study genetic influences on health and disease. Journal of clinical epidemiology. 2016;70:214–223. doi: 10.1016/j.jclinepi.2015.09.016 26441289

[13] Loh PR, Bhatia G, Gusev A, Finucane HK, Bulik-Sullivan BK, Pollack SJ, et al. Contrasting genetic architectures of schizophrenia and other complex diseases using fast variance-components analysis. Nature genetics. 2015;47(12):1385. doi: 10.1038/ng.3431 26523775 PMC4666835

[14] Loh PR. BOLT-LMM v2. 3.2 User Manual. Available oniline at: https://databroadinstitute.org/alkesgroup/BOLT-LMM/ (accessed May 2, 2019). 2018;.

[15] Bulik-Sullivan BK, Loh PR, Finucane HK, Ripke S, Yang J, Patterson N, et al. LD Score regression distinguishes confounding from polygenicity in genome-wide association studies. Nature genetics. 2015;47(3):291. doi: 10.1038/ng.3211 25642630 PMC4495769

[16] Ge T, Reuter M, Winkler AM, Holmes AJ, Lee PH, Tirrell LS, et al. Multidimensional heritability analysis of neuroanatomical shape. Nature communications. 2016;7:13291. doi: 10.1038/ncomms13291 27845344 PMC5116071

[17] Lin Z., Seal S. and Basu S., 2022. Estimating SNP heritability in presence of population substructure in biobank-scale datasets.Genetics,220(4), p.iyac015. 35106569 10.1093/genetics/iyac015 PMC8982037

[18] Speed D, Hemani G, Johnson MR, Balding DJ. Improved heritability estimation from genome-wide SNPs. The American Journal of Human Genetics. 2012;91(6):1011–1021. doi: 10.1016/j.ajhg.2012.10.010 23217325 PMC3516604

[19] Speed D, Balding DJ. MultiBLUP: improved SNP-based prediction for complex traits. Genome research. 2014;24(9):1550–1557. doi: 10.1101/gr.169375.113 24963154 PMC4158754

[20] Speed D, Cai N, Johnson MR, Nejentsev S, Balding DJ. Reevaluation of SNP heritability in complex human traits. Nature genetics. 2017;49(7):986–992. doi: 10.1038/ng.3865 28530675 PMC5493198

[21] Zhang Q, Privé F, Vilhjálmsson B, Speed D. Improved genetic prediction of complex traits from individual-level data or summary statistics. bioRxiv. 2021; p. 2020–08. 10.1038/s41467-021-24485-y PMC8263809 34234142

[22] Kingman JF. Origins of the coalescent: 1974-1982. Genetics. 2000;156(4):1461–1463. doi: 10.1093/genetics/156.4.1461 11102348 PMC1461350

[23] Degnan JH, Salter LA. Gene tree distributions under the coalescent process. Evolution. 2005;59(1):24–37. doi: 10.1111/j.0014-3820.2005.tb00891.x 15792224

[24] Banerjee S, Gelfand AE, Finley AO, Sang H. Gaussian predictive process models for large spatial data sets. Journal of the Royal Statistical Society: Series B (Statistical Methodology). 2008;70(4):825–848. doi: 10.1111/j.1467-9868.2008.00663.x 19750209 PMC2741335

[25] Finley AO, Sang H, Banerjee S, Gelfand AE. Improving the performance of predictive process modeling for large datasets. Computational statistics & data analysis. 2009;53(8):2873–2884. doi: 10.1016/j.csda.2008.09.008 20016667 PMC2743161

[26] Zhou X, Stephens M. Efficient multivariate linear mixed model algorithms for genome-wide association studies. Nature methods. 2014;11(4):407. doi: 10.1038/nmeth.2848 24531419 PMC4211878

[27] Rosenberg NA, Nordborg M. Genealogical trees, coalescent theory and the analysis of genetic polymorphisms. Nature Reviews Genetics. 2002;3(5):380–390. doi: 10.1038/nrg795 11988763

[28] Basseville M, Benveniste A, Chou KC, Golden SA, Nikoukhah R, Willsky AS. Modeling and estimation of multiresolution stochastic processes. IEEE Transactions on Information Theory. 1992;38(2):766–784. doi: 10.1109/18.119735

[29] Jiang J, Li C, Paul D, Yang C, Zhao H, et al. On high-dimensional misspecified mixed model analysis in genome-wide association study. The Annals of Statistics. 2016;44(5):2127–2160. doi: 10.1214/15-AOS1421

[30] Pritchard JK, Przeworski M. Linkage disequilibrium in humans: models and data. The American Journal of Human Genetics. 2001;69(1):1–14. doi: 10.1086/321275 11410837 PMC1226024

[31] Bradley RC. Basic properties of strong mixing conditions. A survey and some open questions. arXiv preprint math/0511078. 2005;.

[32] Mokkadem A. Mixing properties of ARMA processes. Stochastic processes and their applications. 1988;29(2):309–315. doi: 10.1016/0304-4149(88)90045-2

[33] Nobel A, Dembo A. A note on uniform laws of averages for dependent processes. Statistics & Probability Letters. 1993;17(3):169–172. doi: 10.1016/0167-7152(93)90163-D

[34] Heaton MJ, Datta A, Finley AO, Furrer R, Guinness J, Guhaniyogi R, et al. A case study competition among methods for analyzing large spatial data. Journal of Agricultural, Biological and Environmental Statistics. 2019;24(3):398–425. doi: 10.1007/s13253-018-00348-w 31496633 PMC6709111

[35] Eaton ML. Multivariate statistics: a vector space approach. JOHN WILEY & SONS, INC, 605 THIRD AVE, NEW YORK, NY 10158, USA, 1983, 512. 1983;.

[36] Gentle JE. Matrix algebra. Springer texts in statistics, Springer, New York, NY, doi. 2007;10:978–0.

[37] Riedel KS. A Sherman–Morrison–Woodbury identity for rank augmenting matrices with application to centering. SIAM Journal on Matrix Analysis and Applications. 1992;13(2):659–662. doi: 10.1137/0613040

[38] Harville DA. Matrix algebra from a statistician’s perspective; 1998.

[39] Gelfand AE, Banerjee S, Finley AO. Spatial design for knot selection in knot-based dimension reduction models. Spatio-temporal design: Advances in efficient data acquisition. 2012; p. 142–169. doi: 10.1002/9781118441862.ch7

[40] Balding DJ, Nichols RA. A method for quantifying differentiation between populations at multi-allelic loci and its implications for investigating identity and paternity. Genetica. 1995;96(1-2):3–12. doi: 10.1007/BF01441146 7607457

[41] Price AL, Patterson NJ, Plenge RM, Weinblatt ME, Shadick NA, Reich D. Principal components analysis corrects for stratification in genome-wide association studies. Nature genetics. 2006;38(8):904–909. doi: 10.1038/ng1847 16862161

[42] Michalos AC. Encyclopedia of quality of life and well-being research. Springer Netherlands Dordrecht; 2014.

[43] Galinsky KJ, Loh PR, Mallick S, Patterson NJ, Price AL. Population structure of UK Biobank and ancient Eurasians reveals adaptation at genes influencing blood pressure. The American Journal of Human Genetics. 2016;99(5):1130–1139. doi: 10.1016/j.ajhg.2016.09.014 27773431 PMC5097941

[44] Bycroft C, Freeman C, Petkova D, Band G, Elliott LT, Sharp K, et al. Genome-wide genetic data on 500,000 UK Biobank participants. BioRxiv. 2017; p. 166298.

[45] Hou K, Burch KS, Majumdar A, Shi H, Mancuso N, Wu Y, et al. Accurate estimation of SNP-heritability from biobank-scale data irrespective of genetic architecture. Nature genetics. 2019;51(8):1244–1251. doi: 10.1038/s41588-019-0465-0 31358995 PMC6686906

[46] Ge T, Chen CY, Neale BM, Sabuncu MR, Smoller JW. Phenome-wide heritability analysis of the UK Biobank. PLoS genetics. 2017;13(4):e1006711. doi: 10.1371/journal.pgen.1006711 28388634 PMC5400281

[47] Walters R, Abbott L, Bryant S, Churchhouse C, Palmer D, Neale B. Heritability of> 2,000 traits and disorders in the UK Biobank; 2018.

[48] Yengo L, Sidorenko J, Kemper KE, Zheng Z, Wood AR, Weedon MN, et al. Meta-analysis of genome-wide association studies for height and body mass index in 700000 individuals of European ancestry. Human molecular genetics. 2018;27(20):3641–3649. doi: 10.1093/hmg/ddy271 30124842 PMC6488973

