# Supplementary figures and images for: Efficient estimation of SNP heritability using Gaussian predictive process in large scale cohort studies
Source: PLoS Genet. 2022 Apr 20;18(4):e1010151. doi: 10.1371/journal.pgen.1010151 (PMC9060362; doi:10.1371/journal.pgen.1010151)

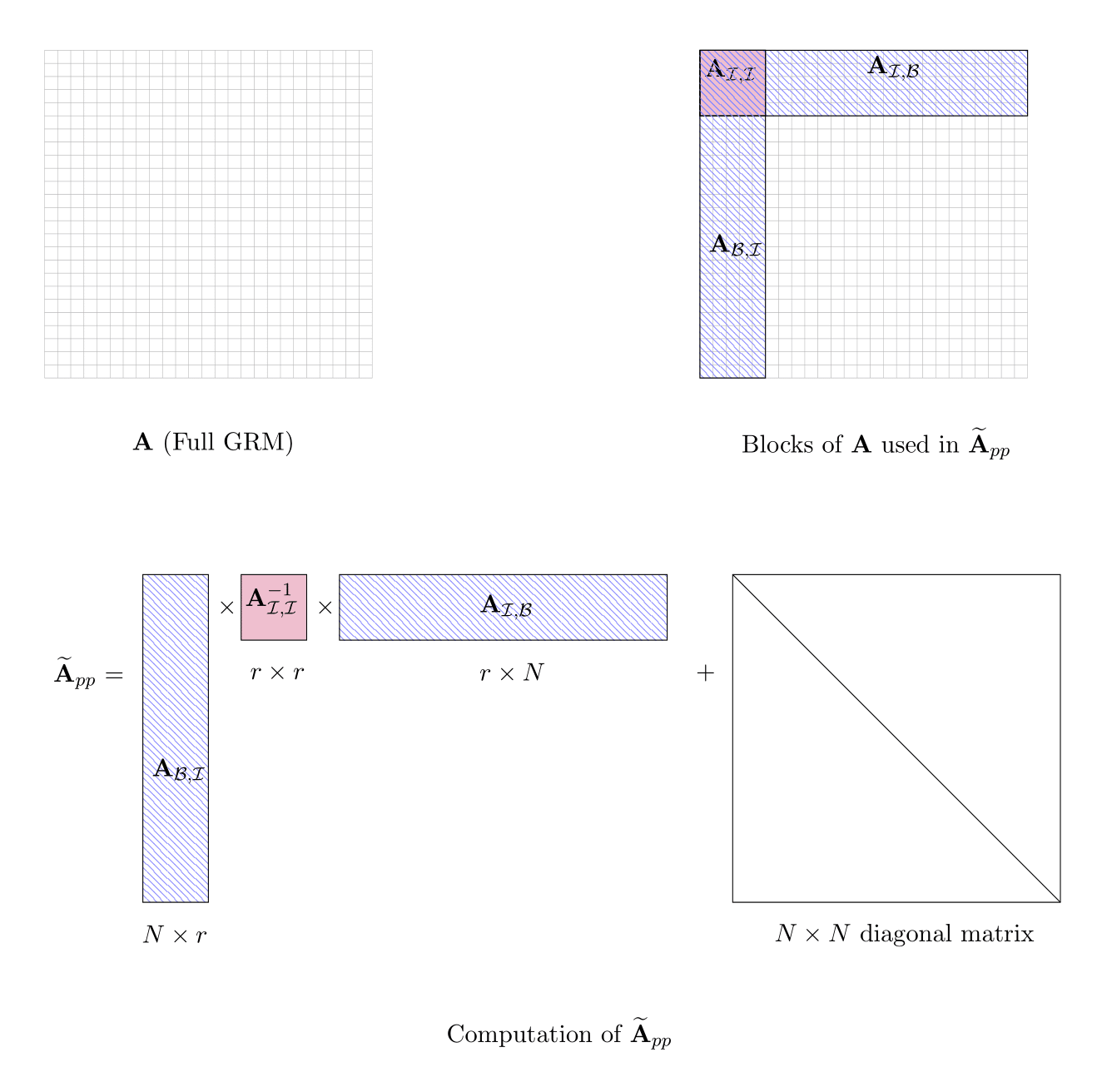

Supplement: S1 Fig — We look at the full GRM A and its blocks that are used in computing A˜PP. For sake of simplicity in representation, we assume that first r of the total of N individuals are in the set of knots I. (TIF) [file pgen.1010151.s001.tif]

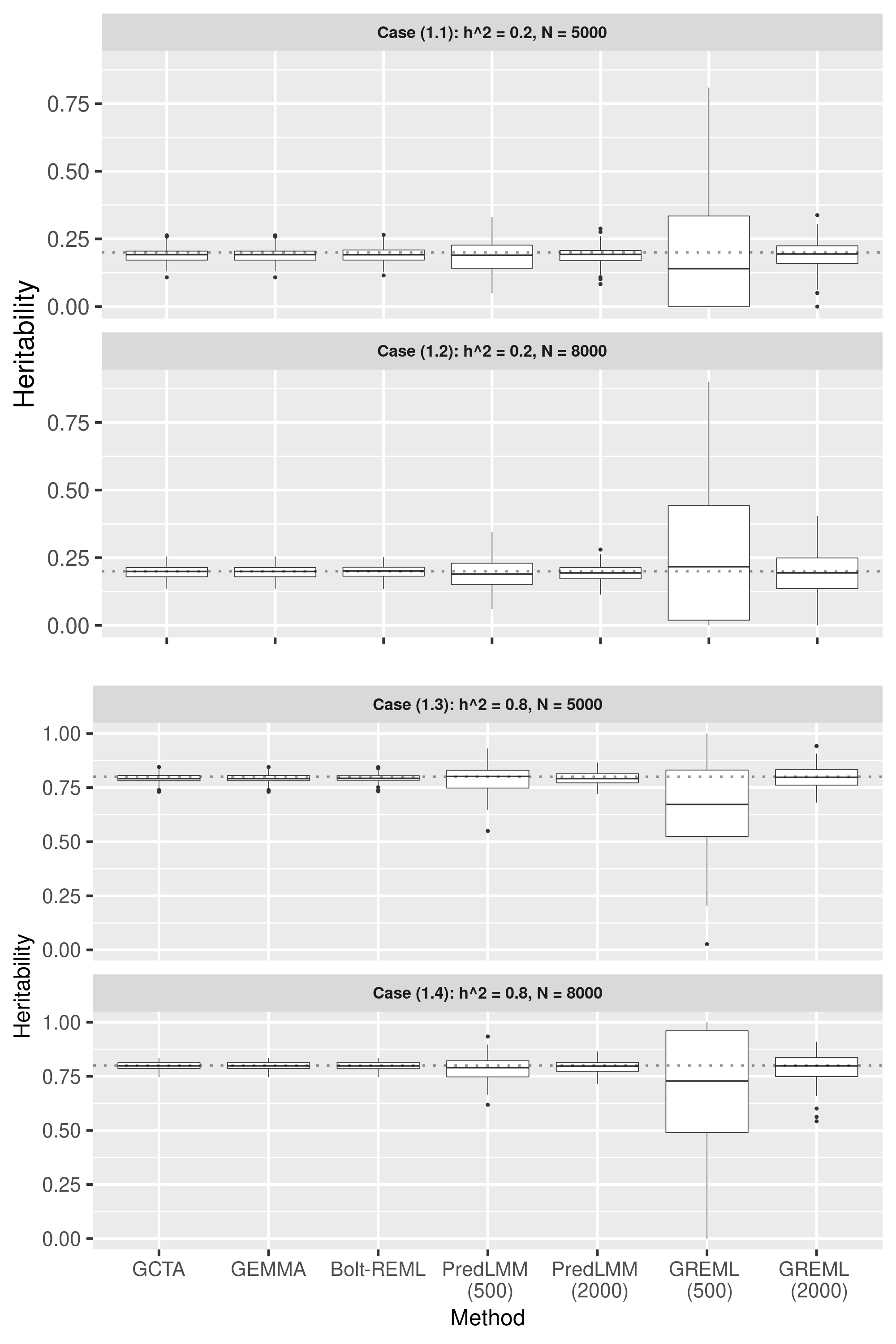

Supplement: S2 Fig — Box-plots of the estimates are shown for varying sub-sample sizes (knot-sizes) in four different cases. (TIF) [file pgen.1010151.s002.tif]

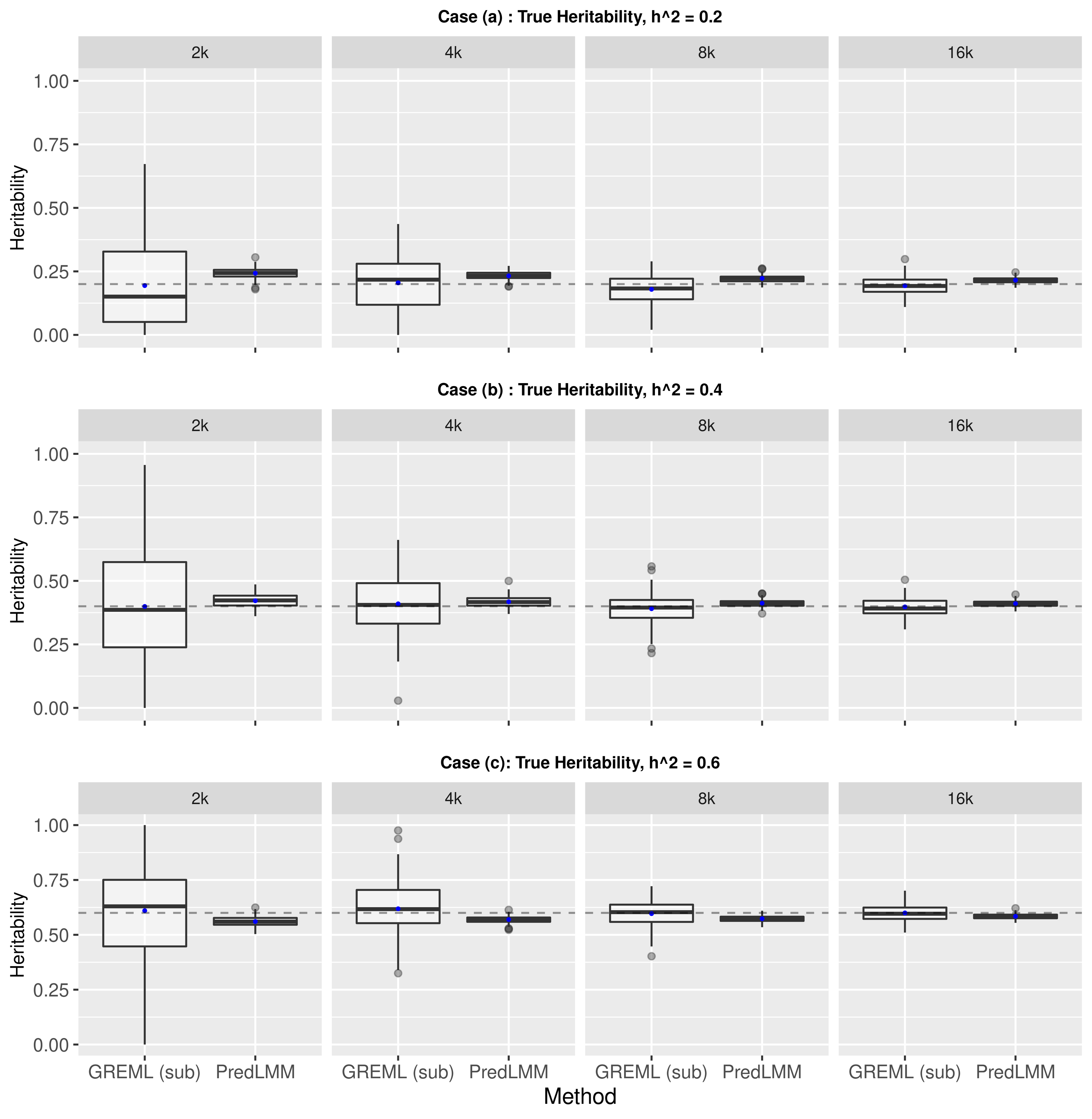

Supplement: S3 Fig — Box-plots of the estimates are shown for varying sub-sample sizes (knot-sizes) in three different cases. (TIF) [file pgen.1010151.s003.tif]

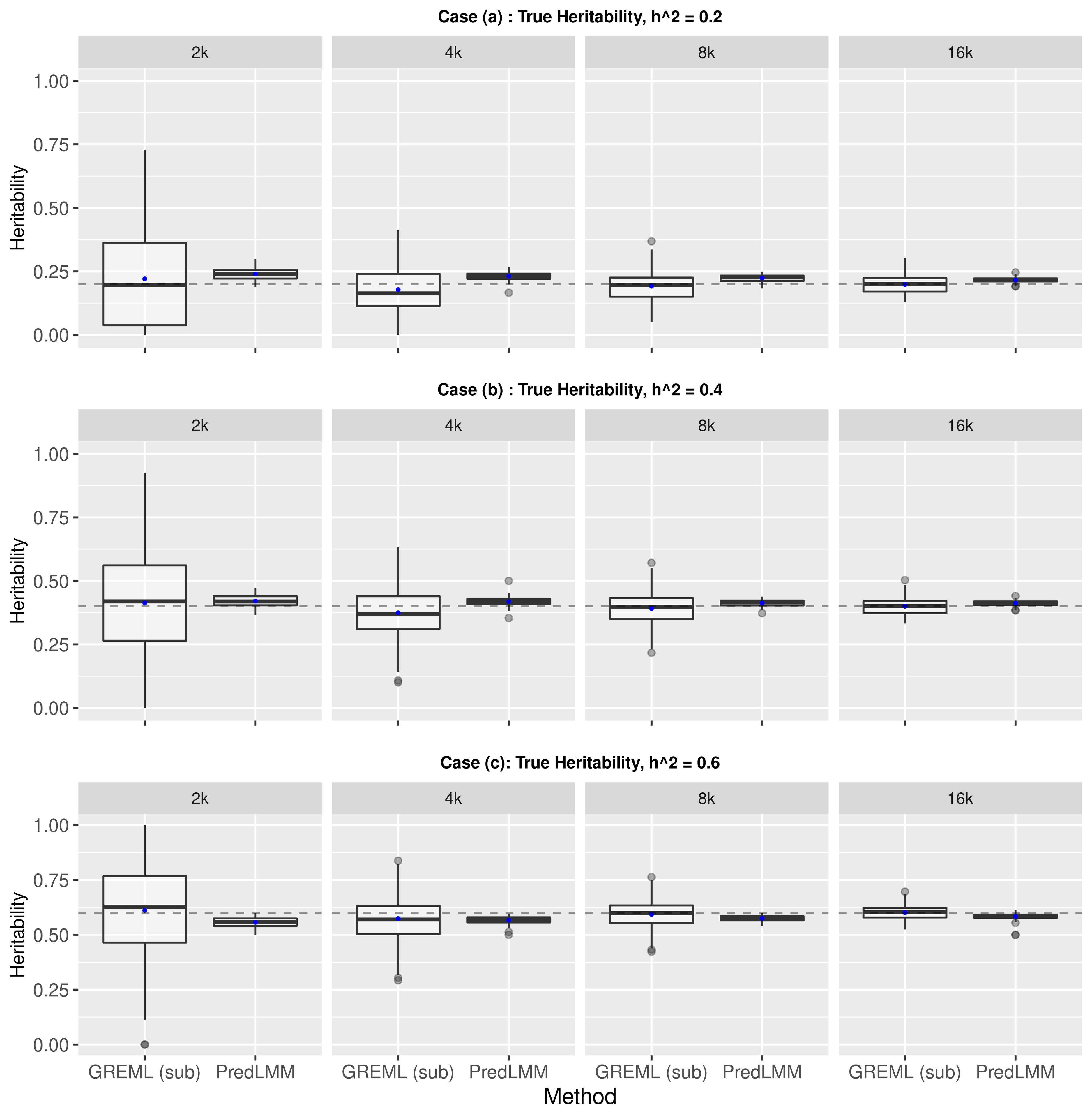

Supplement: S4 Fig — Box-plots of the estimates are shown for varying sub-sample sizes (knot-sizes) in three different cases. (TIF) [file pgen.1010151.s004.tif]
