# Supplementary material for: Efficient estimation of SNP heritability using Gaussian predictive process in large scale cohort studies
Source: PLoS Genet. 2022 Apr 20;18(4):e1010151. doi: 10.1371/journal.pgen.1010151 (PMC9060362; doi:10.1371/journal.pgen.1010151)
Supplement: S1 Appendix — We derive an approximate estimate of the asymptotic variance of the PredLMM estimator. Using the proposed variance formula, we present two tables that list coverage probability of PredLMM under different simulation setups. We also provide the details on the efficient matrix operations that PredLMM makes use of. (PDF) [file pgen.1010151.s005.pdf]

S1 Appendix

Souvik Seal, Abhirup Datta, Saonli Basu

April 10, 2022

Efficient matrix operations involving $\widetilde{\mathbf{A}}_{PP}$

Lets denote the covariance matrix as, $\sigma^2\mathbf{V}^{pp} = \sigma_h^2\widetilde{\mathbf{A}}_{PP} + \sigma_e^2\mathbf{I}$, where $\sigma^2 = \sigma_h^2 + \sigma_e^2$ and $\mathbf{V}^{pp}$ is written as,

$$\mathbf{V}^{pp} = \frac{\sigma_h^2}{\sigma^2}\widetilde{\mathbf{A}}_{PP} + \frac{\sigma_e^2}{\sigma^2}\mathbf{I} = h^2\widetilde{\mathbf{A}}_{PP} + (1-h^2)\mathbf{I}$$

where $h^2 = \frac{\sigma_h^2}{\sigma^2}$. Here we discuss how Woodbury matrix identity [1] can be used to compute the inverse of $\mathbf{V}^{pp}$ with computational complexity of $O(Nr^2 + r^3)$.

$$\begin{aligned}
\mathbf{V}^{pp-1} &= (h^2\widetilde{\mathbf{A}}_{PP} + (1-h^2)\mathbf{I})^{-1} \\
&= (h^2\mathbf{A}_{\mathcal{B},\mathcal{I}}\mathbf{A}_{\mathcal{I},\mathcal{I}}^{-1}\mathbf{A}_{\mathcal{I},\mathcal{B}} + h^2 diag(\mathbf{A}_{\mathcal{B},\mathcal{B}} - \mathbf{A}_{\mathcal{B},\mathcal{I}}\mathbf{A}_{\mathcal{I},\mathcal{I}}^{-1}\mathbf{A}_{\mathcal{I},\mathcal{B}}) + (1-h^2)\mathbf{I})^{-1} \\
&= (h^2\mathbf{A}_{\mathcal{B},\mathcal{I}}\mathbf{A}_{\mathcal{I},\mathcal{I}}^{-1}\mathbf{A}_{\mathcal{I},\mathcal{B}} + E)^{-1} \\
&= E^{-1} - E^{-1}\mathbf{A}_{\mathcal{B},\mathcal{I}}\left(\frac{1}{h^2}\mathbf{A}_{\mathcal{I},\mathcal{I}} + \mathbf{A}_{\mathcal{I},\mathcal{B}}E^{-1}\mathbf{A}_{\mathcal{B},\mathcal{I}}\right)^{-1}\mathbf{A}_{\mathcal{I},\mathcal{B}}E^{-1}
\end{aligned}$$

where $E = h^2 diag(\mathbf{A}_{\mathcal{B},\mathcal{B}} - \mathbf{A}_{\mathcal{B},\mathcal{I}}\mathbf{A}_{\mathcal{I},\mathcal{I}}^{-1}\mathbf{A}_{\mathcal{I},\mathcal{B}}) + (1-h^2)\mathbf{I}$ is a diagonal matrix. Since E is diagonal, computing its inverse E^{-1} takes complexity of $O(N)$. Computing $E^{-1}\mathbf{A}_{\mathcal{B},\mathcal{I}}$ takes complexity of $O(Nr)$. Computing the product of $\mathbf{A}_{\mathcal{I},\mathcal{B}}$ and $E^{-1}\mathbf{A}_{\mathcal{B},\mathcal{I}}$ takes complexity of $O(Nr^2)$. $(\mathbf{A}_{\mathcal{I},\mathcal{I}}+\mathbf{A}_{\mathcal{I},\mathcal{B}}E^{-1}\mathbf{A}_{\mathcal{B},\mathcal{I}})$ is a $r \times r$ matrix computing whose inverse takes complexity $O(r^3)$. Finally, computing the product of $E^{-1}\mathbf{A}_{\mathcal{B},\mathcal{I}}$, $(\mathbf{A}_{\mathcal{I},\mathcal{I}} + \mathbf{A}_{\mathcal{I},\mathcal{B}} + E^{-1}\mathbf{A}_{\mathcal{B},\mathcal{I}})^{-1}$ and $\mathbf{A}_{\mathcal{I},\mathcal{B}}E^{-1}$ takes up complexity of $O(Nr^2)$ (since the matrices have dimensions $N \times r$, $r \times r$ and $r \times N$ respectively).

Variance of PredLMM estimator

Here, we derive an approximate estimate of the asymptotic variance of the PredLMM estimator. PredLMM considers the following multivariate normal distribution, $\mathbf{Y} \sim N_N(\mathbf{X}\boldsymbol{\beta}, \sigma^2\mathbf{V}^{pp})$. $\mathbf{V}^{pp}$ can also be written as,

$$\mathbf{V}^{pp} = h^2\widetilde{\mathbf{A}}_{PP} + (1-h^2)\mathbf{I} = h^2(\widetilde{\mathbf{A}}_{PP} - \mathbf{I}) + \mathbf{I} = h^2\mathbf{A}^{pp,c} + \mathbf{I}$$

where $\mathbf{A}^{pp,c} = (\widetilde{\mathbf{A}}_{PP} - \mathbf{I})$. Lets denote the corresponding log likelihood by $l^{pp}(\mathbf{Y})$. The first derivative of $l^{pp}(\mathbf{Y})$ w.r.t $\boldsymbol{\beta}, \sigma^2, h^2$ would be,

$$\frac{\partial l^{pp}(\mathbf{Y})}{\partial \boldsymbol{\beta}} = \frac{\mathbf{X}^T \mathbf{V}^{pp^{-1}}(\mathbf{Y} - \mathbf{X}\boldsymbol{\beta})}{\sigma^2}; \quad \frac{\partial l^{pp}(\mathbf{Y})}{\partial \sigma^2} = \frac{1}{2}\left(\frac{N}{\sigma^2} - \frac{(\mathbf{Y} - \mathbf{X}\boldsymbol{\beta})^T \mathbf{V}^{pp-1}(\mathbf{Y} - \mathbf{X}\boldsymbol{\beta})}{\sigma^4}\right)$$

$$\frac{\partial l^{pp}(\mathbf{Y})}{\partial h^2} = \frac{1}{2}\left(\text{tr}\mathbf{V}^{pp^{-1}}\mathbf{A}^{pp,c} - \frac{(\mathbf{Y} - \mathbf{X}\boldsymbol{\beta})^T \mathbf{V}^{pp^{-1}}\mathbf{A}^{pp,c}\mathbf{V}^{pp^{-1}}(\mathbf{Y} - \mathbf{X}\boldsymbol{\beta})}{\sigma^2}\right)$$

These three equations can be solved iteratively by Newton-Raphson method [2]. $\boldsymbol{\beta}$ and σ^2 both will have closed form solutions at every iteration (t) obtained by equating the respective first derivatives to 0: $\hat{\boldsymbol{\beta}}_{t+1} = (\mathbf{X}^T \hat{\mathbf{V}}_t^{pp^{-1}} \mathbf{X})^{-1} \mathbf{X}^T \hat{\mathbf{V}}_t^{pp^{-1}} \mathbf{Y}$, $\hat{\sigma}^2_{t+1} = \frac{(\mathbf{Y} - \mathbf{X}\hat{\boldsymbol{\beta}}_t)^T \hat{\mathbf{V}}_t^{pp^{-1}} (\mathbf{Y} - \mathbf{X}\hat{\boldsymbol{\beta}}_t)}{N}$ where $\hat{\mathbf{V}}_t^{pp} = \hat{h}_t^2 \widetilde{\mathbf{A}}^{pp} + (1 - \hat{h}_t^2)\mathbf{I}$ and $\hat{h}_t^2$ is the value of h^2 at the t-th iteration.

The actual expression of the asymptotic variance of the estimator of h^2 will involve the information matrices corresponding to parameters $\boldsymbol{\beta}, \sigma^2$. We approximate the asymptotic variance by making a simplifying assumption. Upon estimating the values of $\boldsymbol{\beta}, \sigma^2$, we treat them as known, thereby ignoring their distributional properties. The second derivative of $l^{pp}(\mathbf{Y})$ with respect to h^2 would be,

$$\frac{\partial^2 l^{pp}(\mathbf{Y})}{\partial h^2 \partial h^2} = \frac{1}{2}\left(\text{tr}\mathbf{V}^{pp^{-1}}\mathbf{A}^{pp,c}\mathbf{V}^{pp^{-1}}\mathbf{A}^{pp,c} - \frac{2(\mathbf{Y} - \mathbf{X}\boldsymbol{\beta})^T \mathbf{V}^{pp^{-1}}\mathbf{A}^{pp,c}\mathbf{V}^{pp^{-1}}\mathbf{A}^{pp,c}\mathbf{V}^{pp^{-1}}(\mathbf{Y} - \mathbf{X}\boldsymbol{\beta})}{\sigma^2}\right)$$

Next, we compute the expectation of several important terms.

$$\mathbf{B} = \mathbb{E}\left(-\frac{\partial^2 l^{pp}(\mathbf{Y})}{\partial h^2 \partial h^2}\right) = -\frac{1}{2}\text{tr}\mathbf{V}^{pp^{-1}}\mathbf{A}^{pp,c}\mathbf{V}^{pp^{-1}}\mathbf{A}^{pp,c} + \text{tr}\mathbf{V}^{pp^{-1}}\mathbf{A}^{pp,c}\mathbf{V}^{pp^{-1}}\mathbf{A}^{pp,c}\mathbf{V}^{pp^{-1}}\mathbf{V}$$

$$\begin{aligned}
\mathbf{C} &= \mathbb{E}\left(\frac{\partial l^{pp}(\mathbf{Y})}{\partial h^2}\frac{\partial l^{pp}(\mathbf{Y})}{\partial h^2}^T\right) \\
&= \frac{1}{4}E\{\text{tr}(\mathbf{V}^{pp^{-1}}\mathbf{A}^{pp,c})^2 - 2\text{tr}(\mathbf{V}^{pp^{-1}}\mathbf{A}^{pp,c})y^T\mathbf{V}^{pp^{-1}}\mathbf{A}^{pp,c}\mathbf{V}^{pp^{-1}}y + (y^T\mathbf{V}^{pp^{-1}}\mathbf{A}^{pp,c}\mathbf{V}^{pp^{-1}}y)^2\} \\
&= \frac{1}{4}\{(\text{tr}(\mathbf{V}^{pp^{-1}}\mathbf{A}^{pp,c})^2 - 2(\text{tr}(\mathbf{V}^{pp^{-1}}\mathbf{A}^{pp,c})\text{tr}(\mathbf{V}^{pp^{-1}}\mathbf{A}^{pp,c}\mathbf{V}^{pp^{-1}}\mathbf{V}) \\
&+ (\text{tr}(\mathbf{V}^{pp^{-1}}\mathbf{A}^{pp,c}\mathbf{V}^{pp^{-1}}\mathbf{V}))^2 + 2\text{tr}(\mathbf{V}^{pp^{-1}}\mathbf{A}^{pp,c}\mathbf{V}^{pp^{-1}}\mathbf{V}\mathbf{V}^{pp^{-1}}\mathbf{A}^{pp,c}\mathbf{V}^{pp^{-1}}\mathbf{V})\} \\
&= \frac{1}{4}\{(\text{tr}\mathbf{V}^{pp^{-1}}\mathbf{A}^{pp,c}) - \text{tr}(\mathbf{V}^{pp^{-1}}\mathbf{A}^{pp,c}\mathbf{V}^{pp^{-1}}\mathbf{V}))^2 + 2\text{tr}(\mathbf{V}^{pp^{-1}}\mathbf{A}^{pp,c}\mathbf{V}^{pp^{-1}}\mathbf{V}\mathbf{V}^{pp^{-1}}\mathbf{A}^{pp,c}\mathbf{V}^{pp^{-1}}\mathbf{V})\}
\end{aligned}$$

We have used the fact that the true distribution of $\mathbf{Y}$ is multivariate normal with mean $\mathbf{X}\boldsymbol{\beta}$ and covariance matrix $\sigma^2\mathbf{V} = \sigma_h^2\mathbf{A} + \sigma_e^2\mathbf{I}$ and hence, $\mathbb{E}(\mathbf{Y} - \mathbf{X}\boldsymbol{\beta})(\mathbf{Y} - \mathbf{X}\boldsymbol{\beta})^T = \sigma^2\mathbf{V}$. Then, the asymptotic variance of the PredLMM estimator would be $\mathbf{B}^{-1}\mathbf{C}\mathbf{B}^{-1}$ [3]. Since, the expressions of $\mathbf{B}$ and $\mathbf{C}$ require dense matrix multiplications that can be computationally intractable, it is necessary to reasonably simplify them. We assume that $\mathbf{V}_{pp} \approx \mathbf{V}$ and it leads to, $\mathbf{B} = \mathbf{C} = \frac{1}{2}\text{tr}\mathbf{V}^{pp^{-1}}\mathbf{A}^{pp,c}\mathbf{V}^{pp^{-1}}\mathbf{A}^{pp,c}$. Finally, the variance formula reduces to,

$$\mathbf{B}^{-1}\mathbf{C}\mathbf{B}^{-1} = \mathbf{B}^{-1} = \frac{2}{\text{tr}\mathbf{V}^{pp^{-1}}\mathbf{A}^{pp,c}\mathbf{V}^{pp^{-1}}\mathbf{A}^{pp,c}}$$

We have verified the validity of our variance estimate by investigating the coverage probability in the simulation studies. Table 1 lists the coverage probability of PredLMM

in cases (1.1) and (1.2) from Simulation Study 1 (of the main manuscript) for different knotsizes at level 0.05. Table 2 lists the coverage probability of PredLMM in case (b) from Simulation Study 2A (of the main manuscript) for different knotsizes at level 0.05.

Case	Knot size	Coverage Probability
(1.1)	500	0.95
(1.1)	2000	0.92
(1.2)	500	0.93
(1.2)	2000	0.94

Table 1: The table lists the coverage probability of PredLMM in cases (1.1) and (1.2) from Simulation Study 1 for different knot-sizes at level 0.05.

Knot size	Coverage Probability
2k	0.91
4k	0.92
8k	0.89

Table 2: The table lists the coverage probability of PredLMM in case (b) from Simulation Study 2A for different knotsizes at level 0.05.

References

[1] Kurt S Riedel. A sherman–morrison–woodbury identity for rank augmenting matrices with application to centering. *SIAM Journal on Matrix Analysis and Applications*, 13(2):659–662, 1992.

[2] Tjalling J Ypma. Historical development of the newton–raphson method. *SIAM review*, 37(4):531–551, 1995.

[3] Dennis D Boos and Leonard A Stefanski. *Essential statistical inference: theory and methods*, volume 120. Springer Science & Business Media, 2013.
